# Determination of Cutoff Values for the Screening of Osteosarcopenia in Obese Postmenopausal Women

**DOI:** 10.1155/2021/6634474

**Published:** 2021-03-18

**Authors:** Nurdiana Z. Abidin, Soma R. Mitra

**Affiliations:** ^1^School of Biosciences, Faculty of Science and Engineering, University of Nottingham Malaysia, 43500 Semenyih, Selangor, Malaysia; ^2^Lifestyle Science Cluster, Advanced Medical and Dental Institute, Universiti Sains Malaysia, 13200 Bertam, Pulau Pinang, Malaysia

## Abstract

Osteosarcopenic obesity (OSO) describes the concurrent presence of obesity, low bone mass, and low muscle mass in an individual. Currently, no established criteria exist to diagnose OSO. We hypothesized that obese individuals require different cut-points from standard cut-points to define low bone mass and low muscle mass due to their higher weight load. In this study, we determined cutoff values for the screening of osteosarcopenia (OS) in obese postmenopausal Malaysian women based on the measurements of quantitative ultrasound (QUS), bioelectrical impedance analysis (BIA), and functional performance test. Then, we compared the cutoff values derived by 3 different statistical modeling methods, (1) receiver operating characteristic (ROC) curve, (2) lowest quintile of the study population, and (3) 2 standard deviations (SD) below the mean value of a young reference group, and discussed the most suitable method to screen for the presence of OS in obese population. One hundred and forty-one (*n* = 141) postmenopausal Malaysian women participated in the study. Bone density was assessed using calcaneal quantitative ultrasound. Body composition was assessed using bioelectrical impedance analyzer. Handgrip strength was assessed using a handgrip dynamometer, and physical performance was assessed using a modified Short Physical Performance Battery test. ROC curve was determined to be the most suitable statistical modeling method to derive the cutoffs for the presence of OS in obese population. From the ROC curve method, the final model to estimate the probability of OS in obese postmenopausal women is comprised of five variables: handgrip strength (HGS, with area under the curve (AUC) = 0.698 and threshold ≤ 16.5 kg), skeletal muscle mass index (SMMI, AUC = 0.966 and threshold ≤ 8.2 kg/m^2^), fat-free mass index (FFMI, AUC = 0.946 and threshold ≤ 15.2 kg/m^2^), broadband ultrasonic attenuation (BUA, AUC = 0.987 and threshold ≤ 52.85 dB/MHz), and speed of sound (SOS, AUC = 0.991 and threshold ≤ 1492.15 m/s). Portable equipment may be used to screen for OS in obese women. Early identification of OS can help lower the risk of advanced functional impairment that can lead to physical disability in obese postmenopausal women.

## 1. Introduction

Osteosarcopenic obesity (OSO) is a term used to describe the concurrent presence of obesity, low bone mass (osteoporosis), and low muscle mass (sarcopenia) in an individual [1–4]. OSO is an age-related disorder and considered to be the most advanced functional impairment related to bone, muscle, and adiposity [5]. Other possible manifestations of this syndrome include osteopenic obesity (OO) and sarcopenic obesity (SO), where “obesity” is not necessarily confined only to a clinical diagnosis of overweight or obesity but also includes the aspect of fat infiltration in muscle tissue and its impact on the skeleton. With time, both conditions (OO and SO) are likely to result in OSO.

Currently, information about the etiology, prevalence, and long-term effect of OSO on older adults is sparse. One of the important aspects of OSO is the interconnected nature of the syndrome, from its cellular connections to the deterioration of bone (osteopenia/osteoporosis) and muscle (sarcopenia) in excess of adipose tissue (overweight/obesity). Osteopenia/osteoporosis, sarcopenia, and overweight/obesity were once considered as separate conditions and were rarely studied together. However, multiple studies suggest that bone, muscle, and fat are strongly linked [6–8]. Liu et al. [8], for example, had found a significant and positive correlation between muscle mass and whole-body bone mineral density (BMD) in obese women. Interestingly, when it comes to adiposity, the researchers found inflection points between 22 and 40 kg in fat mass and 33–38% of body fat, whereby negative relationships were noted with BMD. Therefore, it is worth studying in depth the interrelationship between the 3 body components and the etiology of OSO in aging population, especially in women after menopause due to their higher risk of osteoporosis and fat retention. The recognition of the syndrome as a single entity is thought to be more physiologically relevant and may help guide a comprehensive treatment plan.

Due to its relatively recent recognition, currently, there are no standard definitions for OSO, leading to a challenge in official diagnosis. In 2016, Ilich, Kelly, and Inglis had introduced a set of diagnostic criteria for OSO in older women which involved physical and functional assessment [1]. The diagnostic criteria for the physical assessment are as follows: (1) T-score for BMD ≤ −1.0 SD at the femoral neck, proximal femur, or lumbar spine, (2) 20th percentile of appendicular lean mass (ALM) for women, with the equation: ALM = −17.4 + 18.3*x* height (m) + 0.16*x* body fat (kg) [9], and (3) fat mass ≥ 32% of body weight for women. All three criteria were required to be assessed using dual-energy X-ray absorptiometry (DXA), which is sophisticated equipment available only in specialized laboratories and hospitals. The functional assessment includes handgrip strength (≤ 20 kg for women) and modified components of short physical performance battery (SPPB) test: one-leg stance: ≤ 16 sec, gait speed: ≤ 0.8 m/sec, and sit-to-stand chair test: ≤ 20 times. It was clear that these diagnostic criteria were designed to identify OSO when it has reached clinically relevant stages for bone loss, muscle loss, and obesity. By the time, OSO was diagnosed in an individual using these criteria; it may have been too late for mitigation of progression of the disease. There was also the question of applicability across populations. These proposed criteria may not be applicable to all population due to factors such as differences in ethnicity and/or instrumental feasibility in large-scale epidemiological studies. Due to the progressive nature of OSO, early diagnosis of the syndrome is important for effective intervention. Therefore, the aim of the current study was to identify screening test criteria and cutoff values for OSO that are feasible for epidemiological study and screening purposes, while still being accurately predictive of the development of the syndrome by using portable qualitative ultrasound (QUS) machine and other tests that are feasible to be conducted in the community at large. In addition, the current study also compared and contrasted the cutoff values derived by 3 different statistical modeling methods, (1) ROC curve, (2) lowest quintile of the study population, and (3) 2 SD below the mean value of a young reference group, and discussed the most suitable method to screen for the presence of OS among obese postmenopausal women.

## 2. Materials and Methods

### 2.1. Selection and Recruitment of Participants

One hundred and forty-one (*n* = 141) postmenopausal Malaysian women (aged 45 to 88 years) were recruited from various places around the area of Semenyih and Klang Valley, Kuala Lumpur, Malaysia (i.e., Malaysia Menopause Society, senior citizens clubs, residential areas, and religious centers). Postmenopausal was defined as having no menstrual period, bleeding, or spotting during the 12 consecutive months prior to enrolment. Before enrolment, details about the study covering the objectives, procedures, benefits, risks, and possible discomforts from the study were briefed to interested participants. Apparently healthy and interested participants were screened for eligibility with the following inclusion criteria: (i) woman, (ii) citizen of Malaysia (of Malay, Indian, or Chinese ethnicity), and (iii) postmenopausal (no menstrual period, bleeding, or spotting 12 consecutive months prior to enrolment). Exclusion criteria include (i) inability to stand for height, weight, and gait speed assessments, (ii) presence of artificial limbs and/or metal implants, (iii) severe cardiac, pulmonary, or musculoskeletal disorders, (iv) severe cognitive impairment or any disability that makes communication impossible, and (v) presence of terminal illness. Young adult females (*n* = 118, aged 18 to 32 years) were recruited from the University of Nottingham Malaysia to obtain their Est. BMD data in order to generate the T-scores and serve as a reference group. They were also recruited because one of the statistical modeling methods to achieve cut-points requires the mean values from young adults (i.e., two SD below the mean value of a young reference group).

## 3. Demographic Measurements

### 3.1. Demographic Status

Demographic information was collected using a structured and validated questionnaire with items including age, sex, level of education, and history of diseases/comorbidities. Questions on menstrual status were taken from the Menopause Health Questionnaire, The North American Menopause Society (e.g., “how would you describe your current menstrual status?” with options to choose premenopause, perimenopause, and postmenopause, each provided with definitions).

## 4. Diagnostic Measurements

### 4.1. Anthropometric and Obesity Index Measurements

#### 4.1.1. Height

Height was measured to the nearest 0.1 cm using a portable stadiometer (SECA 217, Vogel & Halke GmbH & Co., Hamburg, Germany). Participants were asked to stand with their shoulders, buttocks, and heels resting against the stadiometer, toe tips forming a 45° angle, heels touching each other, head held straight, and neck in a natural position.

#### 4.1.2. Body Fat Percentage and Body Mass Index

Body fat percentage and body mass index were assessed using a segmental bioelectrical impedance analyzer (InBody 230 Body Composition Analyzer, Biospace Co. Ltd., Seoul, Korea), while on this machine, the weight of the participant was automatically generated.

#### 4.1.3. Waist Circumference

A measuring tape (SECA 203, GmbH & Co. Kg., Hamburg, Germany) was used to measure waist circumference. Waist circumference (cm) was measured at the midpoint between the last rib and the anterior superior iliac spine with subjects standing upright.

### 4.2. Bone Density Index Measurement

#### 4.2.1. Quantitative Ultrasound (QUS) Bone Assessments

Bone density was assessed using a calcaneal ultrasound bone densitometer (SAHARA® Clinical Bone Sonometer, Hologic Inc, Waltham, MA, USA). Prior studies using quantitative ultrasound (QUS) found that high-frequency sound waves were attenuated easier by bone compared to low-frequency sound waves. Ultrasonic sound waves in the frequency range of 0.2 to 0.6 MHz were found to be linearly correlated with the level of attenuation. The slope of the linear regression of these two parameters (attenuation versus sound waves in the frequency range) was defined as broadband ultrasound attenuation (BUA) and was measured in dB/MHz. On the SAHARA® system, the BUA and speed of sound (SOS) are measured simultaneously. In order to determine the sound attenuation of the heel alone, without any bias arising from the transducers and/or transducer pads, a comparison measurement must be made through a reference medium. This reference medium was made using the SAHARA® QC Phantom (supplied with the SAHARA® unit) when the unit was calibrated at the factory. The range of BUA observed with SAHARA® in a typical population is approximately 30–130 dB/MHz, with young/healthy subjects having higher BUA results than older or osteoporotic subjects (based on SAHARA® Clinical Bone Sonometer User's Guide, 1998). While SAHARA® densitometer does not directly measure BMD, the BUA and SOS results are correlated (*r* = 0.82–0.85) with heel BMD results obtained by the standard dual-energy X-ray absorptiometry (DXA) technique [10].

### 4.3. Muscle Mass Index Measurements

#### 4.3.1. Skeletal Muscle Mass (SMM), Fat-Free Mass (FFM), and Appendicular Skeletal Muscle Mass (appSMM)

Muscle mass indices were assessed using a segmental bioelectrical impedance analyzer (BIA, InBody 230 Body Composition Analyzer, Biospace Co. Ltd., Seoul, Korea). SMM and FFM were automatically generated by the analyzer. Skeletal muscle mass index (SMMI) was calculated by dividing the value for SMM (kg) by the square of the height (m^2^). Similarly, the fat-free mass index (FFMI) was calculated by dividing the value for FFM (kg) by the square of the height (m^2^). Appendicular skeletal muscle mass was calculated by adding the sum of the muscle masses of the four limbs. Appendicular skeletal muscle mass index (appSMMI) was defined as the sum of the muscle masses of the four limbs, adjusted for height in squared meters (kg)/height^2^. The cutoff criteria for appSMMI, when BIA was used, are ≤ 5.7 kg/m^2^ for women, as recommended by the Asian Working Group on Sarcopenia (AWGS) [11]. AppSMMI was first suggested by Baumgartner et al. [12] in the New Mexico Elder Health Survey. This index provided significant associations with physical disability or frailty.

#### 4.3.2. Handgrip Strength (HGS)

HGS was assessed as a proxy for muscle strength and was measured in each hand using a hand dynamometer (JAMAR Hydraulic Hand Dynamometer® Model PC-5030 J1, Fred Sammons, Inc., Burr Ridge, IL, USA). Handgrip strength was measured twice for each hand, and the higher of the two values was recorded. Then, the higher value of the two hands was used in the analysis. Standardized positioning recommended by the American Society of Hand Therapists (ASHT) was used: subject seated, shoulders adducted and neutrally rotated, elbow flexed at 90°, forearm in neutral, and wrist between 0 and 30° of dorsiflexion [13].

### 4.4. Functional Performance Assessment

#### 4.4.1. Short Physical Performance Battery (SPPB) Test

Functional performance was assessed using modified components of the short physical performance battery (SPPB) test. Based on the recommendation by Ilich, Kelly, and Inglis [1], the following tests were conducted under the SPPB: one-leg stance (to test balance), gait speed (to test endurance), and sit-to-stand chair test (to assess lower extremity strength). The SPPB has an internal consistency of 0.76 and has predictive validity for the risk of mortality, nursing home admission, and disability [14].

*(1) One-Leg Stance*. For the one-leg stance, measurements for both the right and left legs were assessed. The test requires participants to stand on one leg while lifting the other limb, for a maximum of 30 s. The test stops when the participant touches any surface or lowers the other limb to the ground or, ultimately, at the end of 30 s [1].

*(2) Gait Speed*. Gait speed was measured by timing a 6 m normal walk. The 6 m course was marked by two cones or pieces of tape measured using a roll-up, self-retracting construction measuring tape. The test requires the participant to walk at a normal pace starting at one end of the course and all the way past the other. The timing starts when the tester/instructor commands “begin” and stops when one of the participant's feet is all the way across the 6 m marker. If the participants normally use a cane or any other walking devices, they were allowed to use them while performing the test.

*(3) Sit-to-Stand Chair Test*. At the beginning of this test, the participant was seated in an armless chair, with arms crossed over the chest, back straight, and feet flat on the floor. At the maximum of 30 s, the test requires the participant to rise from the chair and sit down again as many times as possible. The number of consecutive chair sit-to-stand tests completed was recorded, with the last time the participant sat down in the chair being the final count.

## 5. Statistical Analysis

Statistical analyses were performed using the statistical program SPSS (version 24 for Windows; SPSS, Inc., Chicago, IL, USA). The variables were checked for normality (Shapiro–Wilk test) and presented as mean ± standard deviation, unless noted otherwise. The characteristics of the study participants were presented as mean and standard deviations (SD) or the number of participants and the corresponding proportion. Frequency and percentages were reported for categorical variables. A comparison of the distributions of various parameters between groups was performed using an ANOVA (analysis of variance) or ANOVA's Welch test. When significant differences were found with ANOVA, the post hoc Tukey's HSD (honestly significant difference) or Games-Howell test was applied to correct for use of multiple comparisons. Two-tailed *p*-value ≤ 0.05 was recognized as statistically significant unless otherwise noted.

### 5.1. Statistical Modeling Methods

#### 5.1.1. Receiver Operating Characteristic (ROC) Curve

Receiver operating characteristic (ROC) curve was used to define optimal cutoff values based on the point closest to 0,1 corner in the ROC plane, which defines the optimal cut-point as the point minimizing the Euclidean distance between the ROC curve and the (0,1) point [15]. Whenever there is a trade-off between sensitivity and specificity, sensitivity was prioritized over specificity to detect screening test criteria [16]. The sensitivity (true positive) represents the proportion of subjects actually presenting with osteosarcopenia (OS), having been correctly identified as OS. The specificity is the proportion of subjects who do not actually have OS, which was incorrectly identified as OS using the screening equipment (false positive). The area under each respective ROC curve (AUC) closest to 1 (> 0.6) is considered as a good predictor (high screening power). The ROC curves in predicting OS were plotted against healthy, obese-only (OB), and counterparts (without osteosarcopenia).

#### 5.1.2. Lowest Quintile (20th)

For this method, the cutoff value for each variable was derived by separating data into quintiles using SPSS, and the cutoff value for the lowest quintile was chosen for the criterion. A quintile is a statistical value of a data set that represents 20% of a given population. The first quintile represents the lowest fifth of data and the final quintile represents the final or last fifth of data. For this method, only healthy participants were included in the analysis. Therefore, postmenopausal women who reported to have been diagnosed with musculoskeletal-related disorders were excluded from the analysis (i.e., osteoarthritis, rheumatoid arthritis, and osteoporosis).

#### 5.1.3. Two SD below the Mean Value of a Young Reference Group

For this method, the cutoff values were derived by subtracting 2SD values from the mean of the young reference group, for each variable of interest. Similarly, for this analysis, postmenopausal women who reported to have been diagnosed with musculoskeletal-related disorders were excluded from the analysis (i.e., osteoarthritis, rheumatoid arthritis, and osteoporosis).

## 6. Ethics

This study was reviewed and approved by the Science and Engineering Research Ethics Committee of the University of Nottingham Malaysia (SEREC-NZA051016). In accordance with the Helsinki Declaration, before entering the study, each subject gave informed written consent.

## 7. Results

Characteristics of participants are presented in Table 1. Participants were categorized into “OSO,” “OO,” “SO,” “OB,” and “NR” groups based on the criteria and standard cutoff proposed by previous studies, WHO (T-score ≤ 2.5) [17], Ilich et al. (BFP ≥ 32%) [1], and the Asian Working Group for Sarcopenia (AWGS, appSMMI ≤ 5.7 kg/m^2^) [11]. In the current study, the T-scores were derived based on QUS-generated Est. BMD of young Malaysian women aged 18–32 years. Differences in the characteristics are depicted in Table 1.

In this sample population, there were 5.7% (*n* = 8) of women with OSO, 4.3% (*n* = 6) of women with OO, and 17.0% (*n* = 24) of women with SO. The majority of participants were obese-only (OB, 58.2%, *n* = 82), and only 5.0% (*n* = 7) of women were normal weight, with healthy bone and muscle mass (NR). Fourteen (*n* = 14, 10%) participants were either sarcopenic-only (normal weight) or osteopenic/osteoporotic-only (normal weight).

The OSO group was significantly older and thinner, with smaller waist circumference and lower muscle mass compared to OB participants (*p* < 0.05). Years since menopause had no impact on any of the disorders. Obese participants (with and without the musculoskeletal disorder) had significantly higher body fat percent (BFP) and trunk fat percent and significantly weaker handgrip strength compared to the normal-weight participants (NR) (*p* < 0.001).

Interestingly, although OB and NR groups have a similar amount of muscle mass (no significant difference), the OB group had significantly lower handgrip strength compared to the NR group. No significant difference was found for lower extremity strength (sit-to-stand test), endurance (gait speed), and balance between any of the groups (OSO, OO, SO, OB, and NR).

OSO group and SO group did not differ significantly in any of the listed variables (age, anthropometrics, body composition, and physical performance). Conversely, the OO group had significantly higher muscle mass (SMMI and appSMMI) compared to both OSO and SO group (*p* < 0.001). Despite being nonsignificant, the OO group also performed better than OSO and SO for each index of functional performance (handgrip strength (HGS), sit-to-stand test, gait speed, and balance).

Table 2 shows a comparison of characteristics between young and postmenopausal women. For this analysis, postmenopausal women who reported to have been diagnosed with musculoskeletal-related disorders were excluded from the analysis (i.e., osteoarthritis, rheumatoid arthritis, and osteoporosis).

The result shows that the younger age group was significantly younger and taller and have stronger handgrip strength and denser bone compared to their older counterparts (*p* ≤ 0.05). Conversely, older women were found to be significantly heavier (body weight and BMI), with larger midsection (WC) and higher body fat percent (BFP) compared to their younger counterparts (*p* ≤ 0.05). Consequently, their muscle mass indices (FFMI, SMMI, and appSMMI) were also significantly higher than their younger counterparts (*p* ≤ 0.05).

Table 3 shows differences in cutoff values using 3 different methods of analysis. Comparatively, when using the ROC and the lowest 20th percentile method to derive the values, the cutoffs were similar to the standard cutoffs proposed by previous studies [11, 18].

Interestingly, the cutoff values derived using 2SD below the mean of the young reference group were much lower than the standard cutoffs.

**Figure 1:**
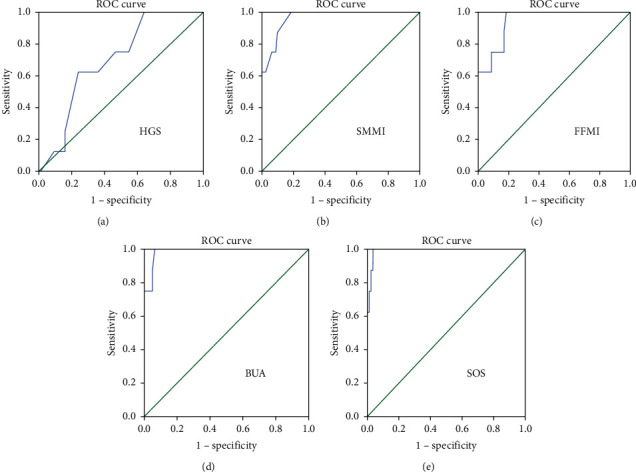
Receiver operating characteristic (ROC) curve showing the ability of handgrip strength (kg), skeletal muscle mass index (kg/m^2^), fat-free mass index (kg/m^2^), broadband ultrasonic attenuation (dB/MHz), and speed of sound (m/sec) to predict osteosarcopenia (T-score ≤ −2.5 and appendicular skeletal muscle mass index ≤ 5.7 kg/m^2^) in obese participants (BFP ≥ 32%, OSO, *N* = 8), plotted against obese participants without osteosarcopenia (T-score > −2.5 and appendicular skeletal muscle mass index > 5.7 kg/m^2^, BFP ≥ 32%, OB, *N* = 82). BFP: body fat percent, OSO: osteosarcopenic obese; OB: obese-only.

Figure 1 depicts the ability of handgrip strength, skeletal muscle mass index, fat-free mass index, broadband ultrasonic attenuation, and speed of sound (HGS, SMMI, FFMI, BUA, and SOS, respectively) in predicting osteosarcopenia (OS) in obese postmenopausal women, using the ROC curve plotted against healthy, obese-only (OB), and counterparts (without osteosarcopenia). These variables were found to be good predictors for the screening of OS in obese women based on their AUC values.

It is interesting to note that the threshold for HGS in the current study was much lower than the threshold proposed by the Asian Working Group for Sarcopenia (AWGS) [11] (16.5 kg versus 18.0 kg, respectively). Additionally, the threshold for BUA was marginally lower than the cutoff for low bone density proposed by Johansen, Evans, and Stone [18] (52.85 versus 54.0 dB/MHz, respectively). Currently, there is no standard threshold for SMMI and FFMI for the screening of sarcopenia in normal-weight or obese individuals.

The values of the AUC for all criteria are shown in Table 4. The optimal cutoffs for the determination of OS in obese postmenopausal Malaysian women were 16.5 kg, 8.2 kg/m^2^, 15.2 kg/m^2^, 52.85 dB/MHz, and 1492.15 m/s for HGS, SMMI, FFMI, BUA, and SOS, respectively.

Table 5 shows the differences in prevalence when the new cutoff values were used to define osteosarcopenic obesity (OSO), osteopenic obesity (OO), sarcopenic obesity (SO), obese-only (OB), and normal (NR) participants.

It is interesting to note that the prevalence for OSO was close to two times higher when SMMI was used as an indicator for muscle mass, compared to FFMI (9.4% versus 5.5%, respectively). The prevalence was also the lowest when functional performance (HGS) was added to the criteria. Out of the 3 sets of new criteria presented in Table 5, Criteria 1 has the highest percentage of sensitivity to screen for OSO.

## 8. Discussion

Osteosarcopenic obesity (OSO) is characterized by the concurrent presence of osteopenia/osteoporosis, sarcopenia, and obesity. In this study, we began by identifying obese participants with musculoskeletal health disorders (OSO, OO, and SO) among community-dwelling, postmenopausal Malaysian women. Then, we evaluated their physical performance and compared them with obese-only (OB) and normal-weight women without musculoskeletal health disorders (NR). Postmenopausal women in this study were categorized into OSO, OO, SO, and OB based on the standard criteria and cutoff proposed by WHO (T-scores ≤ −2.5) [17], the Asian Working Group for Sarcopenia (appSMMI ≤ 5.7 kg/m^2^) [11], and Ilich et al. (BFP ≥ 32%) [1]. Normal-weight participants without musculoskeletal health disorders (NR) were kept as the control group. The majority of studies (∼90%) had used exclusively muscle mass for the definition of sarcopenia, while less than 10% of studies included mass, strength, and functional performance, as recommended by the European Sarcopenia Consensus [19]. For this reason, we decided to use only muscle mass for the definition of sarcopenia in the classification of OSO and its variants in this study.

Findings showed that the majority of women in this sample population was OB at 58.2%, followed by SO at 17.0%, OSO at 5.7%, NR at 5.0%, and finally, OO at 4.3% (Table 1). In contrast to the findings by Ilich et al. [9], the proportion for SO in the current study was higher than OO. There is a valid explanation for this finding. Various studies (including the current study) have found direct and positive correlations between muscle mass and bone density (results presented elsewhere), implying that if one decreases, the other might follow. Muscle loss typically occurs first before bone loss [20]; hence, in theory, the proportion of people with SO in the population should be higher than OO. Conversely, OO, where individuals were obese, with a healthy volume of muscle mass but low bone mass, should in theory be difficult to find in the population due to built-in remodeling function of bones. In the current study, there were only 4.3% of participants with this condition (Table 1).

OSO is a progressive disorder which can begin with any one of the three conditions: osteopenia/osteoporosis, sarcopenia, or obesity. According to Ilich et al. [1], OSO likely occurs due to deregulation of stem cell lineage, which leads to impedance in the cross-talk between bone, muscle, and fat through altered concentrations of osteokine (bone), myokine (muscle), and adipokine (fat). People with OSO tend to have a myriad of detrimental side effects such as a higher risk of falls, fractures, disability, and reduced quality of life [1, 2, 9, 21]. To date, little is known about the prevalence of OSO in the general population, in no small part due to lack of consensus in the test criteria, definitions, and cutoffs of the syndrome components. We had found that the prevalence of OSO in the current study was higher if the T-scores were generated using nonlocal young adults as population of reference (i.e., T-score generated using SAHARA® built-in Hong Kong young females as population of reference = 10.2% versus T-score generated using Malaysian young females as population of reference = 5.7%). Clearly, the cutoff and test criteria for the disorder need to be population-specific. A study by Ilich et al. on Caucasian postmenopausal women in 2015 [9] found that the percentage of OSO in their study population was 12.0% (32 out of 258 postmenopausal women). Comparatively, a Mexican study involving 543 adults found that 16.6% of its study population had OSO [21]. However, in a Korean study on postmenopausal women, the prevalence of OSO in their sample population was higher at 25% [22]. Similarly, Inglis et al. [23] also reported the same percentage of women with OSO (at 25.0%) in a study involving over 500 overweight/obese Caucasian women across the life span. The high margin in various prevalence studies was likely due to differences in the criteria used, ethnic and genetic background, and equipment used, as well as differences in cutoff points and hence the need for standardization in characterizing OSO in the general population.

In the current study, participants in the OSO group were significantly older and slimmer, with a smaller waist and lower muscle mass compared to OB participants (*p* ≤ 0.05), showing the phenotype of “fat frail” (Table 1). Additionally, the OSO group demonstrated significantly lower handgrip strength (HGS), lower muscle mass (whole body and peripheral, Table 1), and bone density (BUA) compared to the NR group (*p* ≤ 0.05). Although no significance was detected, the OSO group in the current study also had lower scores for gait speed, sit-to-stand test, and one-leg stance compared to other groups (SO, OO, OB, and NR). These findings supported the findings by Ilich et al. [9] which showed that OSO syndrome was correlated with low handgrip score, slow normal and brisk walking speed, and short time for leg stance. The results suggest that HGS could be used as additional test criteria for identifying OSO in an individual. Reduced grip strength has been shown to lead to a greater risk of fragility fractures and associated morbidity [24]. Studies have shown that muscle strength is a stronger predictor of long-term functional decline than muscle mass [25]. Yang et al. [26] found that low handgrip strength, combined with high BMI, was strongly associated with an increased risk of functional decline.

### 8.1. Sarcopenic Obesity

Sarcopenic obesity (SO) is described as the concurrent presence of sarcopenia and obesity. SO is a major health concern due to its correlation to reduced activities of daily living (ADL) and increased risk of physical limitations. The combination of high fat and low peripheral muscle mass leads SO to be recognized as “fat frail.” Interestingly, the current study found no significant differences in age, anthropometrics, body composition, and physical performance between the OSO group and SO group (Table 1), indicating that people with SO have a similar degree of physical impairments to OSO. A Korean study involving middle-aged and elderly males and females (aged 50 years or older) found that SO was strongly associated with osteoporosis [27], suggesting close relation between SO and OSO. In general population, the prevalence of SO was hypothesized to be high among the elderly aged 65 years and older due to age-related increases in fat mass and reduced muscle mass [28]. Although it is difficult to compare the prevalence of SO due to differences in populations and the definitions of SO, the approximate average prevalence of SO in older adults was estimated to be about 5–10%, and the prevalence is significantly higher in people aged ≥ 80 years [28].

### 8.2. Osteopenic obesity

There is a marginally lower prevalence of osteopenic obesity (OO) in the population, as obese people, in general, tend to have high bone mineral content and density. In the current study, only 4.3% of the study population had the disorder (Table 1). OO is a combined condition of low bone density and high body fat. Ilich et al. [9] reported that obese women with osteopenia/osteoporosis (OO) had significantly lower physical performance (such as normal walking speed) than obese-only women (those with healthy muscle and bone mass), suggesting the important role of bones in functional performance. In the current study, although no significance was detected, women with OO had weaker balance and handgrip strength compared to OB and NR women (Table 1). It is also fair to note that the OO group had the highest BMI and body fat percent (BFP) among all the groups (OSO, SO, OB, and NR, Table 1). It is likely that the high adiposity of the OO group was one of the reasons for the poor functional performance.

### 8.3. Obesity Paradox

Currently, there is a misleading term called “healthy obese” being used in literature. This term is used due to various studies highlighting the benefits of obesity on health, also known as “obesity paradox.” Obesity paradox is a medical hypothesis which holds that obesity may, counterintuitively, be protective and associated with greater survival in certain groups of people, such as very elderly individuals or those with certain chronic diseases. Some examples of obesity paradox include (1) protective effect of obesity from osteoporosis and (2) increasing evidence that patients, especially elderly, with several chronic diseases and elevated BMI may demonstrate lower all-cause and cardiovascular mortality compared with patients of normal weight [29]. In the current study, the majority of participants were obese with healthy muscle and bone mass (OB = 58.2%, Table 1). These participants can generally be described as “healthy obese.” It is interesting to note that although this group had significantly higher peripheral muscle mass (appSMMI) compared to their normal-weight counterparts (NR), their handgrip strength was significantly lower compared to NR (Table 1). This supports the “quantity versus quality” argument whereby fat-induced muscle mass has a lower quality compared to protein intake and/or resistance training-induced muscle [29]. One of the reasons is likely due to intramuscular fat infiltration, reducing muscle functionality. Some investigators have shown that although fat mass is positively associated with muscle mass, aging is associated with an increase in intramuscular fat by 35.5–74.6% in men and 16.8–50% in women [30]. Therefore, as an individual age, there is a higher chance of fat infiltration between the muscles. Muscle mass is only useful if it is beneficial to functional performance. Findings from the current study showed that the muscle mass of the “healthy obese,” while being high, was not sufficient nor efficient in giving meaningful benefits to grip strength. This supports the theory that obese individuals may require alternative cutoffs or at least a different set of criteria from normal population for the diagnosis of muscle disorders. Further, while OB and NR groups had a similar amount of muscle mass, OB had significantly higher body weight and BFP than the NR group (Table 1). This means that OB had a significantly heavier weight to carry with a similar amount of muscle mass to people with normal weight.

### 8.4. Determining Cutoff Values for the Screening of Osteosarcopenia in Obese Postmenopausal Women Using Bioelectrical Impedance Analysis (BIA) and Quantitative Ultrasound (QUS)

The current definitions of osteosarcopenic obesity (OSO) are based on the individual definitions of osteoporosis, sarcopenia, and obesity. However, questions arise if OSO should be treated as a singular entity and derive cut-points accordingly. Currently, there are no established criteria to define and to properly diagnose OSO, although there have been some preliminary diagnostic criteria proposed [1, 2]. The criteria, however, require the use of sophisticated equipment available only in specialized laboratories and hospitals, i.e., DXA scan. Studies have shown that different definitions of sarcopenia (low muscle mass) are related to different clinical outcomes, especially in aging population. For example, Jang et al. [31], who studied sex-specific distributions of muscle indices adjusted by height, weight, and BMI, found that height-adjusted muscle mass index showed significant association to major health outcomes only in women. Further, anthropometric parameters are affected by ethnic differences, causing researchers to establish population-specific definitions of the decreased muscle mass in various countries. Therefore, determining appropriate cutoff values for osteosarcopenia diagnosis in Asia is critical to ensure accurate diagnosis and device proper treatments specific for the Asian population. Several authoritative research groups have proposed varying definitions of sarcopenia. Persistent controversies exist in how to define reduced skeletal muscle mass. Different cutoffs also exist for different methods of assessment. European Working Group on Sarcopenia in Older People (EWGSOP) recommends DXA, computed tomography (CT), magnetic resonance imaging (MRI), and bioelectrical impedance analysis (BIA) for sarcopenia studies [32]. Although the precision of DXA, CT, and MRI had been well established, when it comes to BIA, some limitations exist in measuring muscle mass. BIA was developed to estimate the volume of body fat and whole-body lean muscle mass. Very few models of BIA were created with a function to measure appendicular lean mass (appSMMI), rendering the use of current diagnostic criteria difficult to be used for screening purposes in the general population (appSMMI ≤ 5.7 kg/m^2^) [11]. In order to limit the use of nonportable and costly diagnostic devices, researchers are striving to develop screening methods to allow clinicians to identify only high-risk individuals to undergo a more demanding diagnostic instrument to determine the presence of sarcopenia. Early identification of sarcopenia will allow the implementation of preventive strategies, thus reducing the risk of fracture and hospitalization.

Currently, the most logistically-friendly and cost-effective screening techniques available for osteoporosis, sarcopenia, and obesity would be quantitative ultrasound-based devices (QUS) and bioelectrical impedance devices (BIA). These devices are portable and the time required for the assessments is short, making them the best devices to be used for screening methods in the general population. For example, calcaneus QUS machines such as the SAHARA^®^ bone densitometer has been found to be good at predicting fragility fracture in postmenopausal women (hip, vertebral, and global fracture risk) and men over the age of 65 (hip and all nonvertebral fractures), independently of central DXA BMD [33].

Sarcopenia working groups such as AWGS (Asian) [11] and EWGSOP (European) [32] had proposed diagnostic classifications and cutoff point identification using various different methods. AWGS recommended 2 SD below the mean value of a young reference group or lowest quintile (20%) of the study population as a cutoff, whereas EWGSOP recommends only the former. Often, population and gender-specific lowest quintile (predictive technique) was used as the cutoff value if population norms of young adults were not available. Other studies had used the ROC curve to derive cutoff points for the diagnosis of sarcopenia [20, 34]. In the current study, we determined the cutoff values for osteosarcopenia using all 3 statistical modeling techniques, (1) ROC curve, (2) lowest quintile of study population, and (3) 2 SD below the mean value of a young reference group, and compared the values with standard cutoffs available from other studies.

#### 8.4.1. Receiver Operating Characteristic (ROC) Curve

In order to develop cutoff values for the screening of OSO as a singular entity, receiver operating characteristic (ROC) analysis was ascertained to be the best and the most appropriate method to use. ROC analysis allowed us to determine the area under the curve (AUC). This type of analysis only furnishes dichotomous results to the screening test, with and without the disease. A criterion of an ideal screening test is to demonstrate reasonably accurate sensitivity and specificity. To determine whether the screening test is positive or negative, we used cutoff values proposed by previous studies as the external criteria to identify obese participants (BFP ≥ 32%) with osteosarcopenia (T-score ≤ −2.5 and appSMMI ≤ 5.7 kg/m^2^). The reference group was obese-only (OB) participants (BFP ≥ 32%) without osteosarcopenia (T-score > −2.5, appSMMI > 5.7 kg/m^2^). The “sensitivity” represents the proportion of subjects actually presenting with osteosarcopenia, having been correctly identified as osteosarcopenic using the screening tool (i.e., positive screening test). The “specificity” is the proportion of subjects who do not actually have osteosarcopenia, which were correctly identified as nonosteosarcopenic using the screening tool (i.e., negative screening test).

All of these proportions were presented with their exact 95% confidence interval (CI) (Table 4). An AUC value under 0.5 reflects no discriminatory power, while an AUC between 0.5 and 1.0 has a high predictive value for clinical testing [35]. An AUC closer to 1 demonstrates a higher screening power and is considered to perform better at distinguishing very well those at risk of osteosarcopenia compared to those not at risk. In the current study, we highlighted an excellent performance of the HGS, SMMI, FFMI, BUA, and SOS to predict osteosarcopenia in obese postmenopausal women (AUC value up to 0.9). The ROC curve showed that HGS (≤ 16.5 kg), FFMI (≤ 15.2 kg/m^2^), SMMI (≤ 8.2 kg/m^2^), BUA (≤ 52.85 dB/MHz), and SOS (≤ 1492.15 m/s (Figure 1) were the best markers for the screening of osteosarcopenia. The cutoff values were identified by maximizing the sum of sensitivity and specificity derived on the basis of the persistent lower extremity limitation outcome. When the new cutoff values were used to determine OSO (BFP ≥ 32%, SMMI ≤ 8.2 kg/m^2^, and BUA ≤ 52.85 dB/MHz), the prevalence increased 1.5 times in the sample group (Table 5). This finding corresponds to the finding obtained by Kim et al. [20] who reported the increase of likelihood by 1.88 times when their ROC-derived cutoff values were used to predict the risk of osteoporosis in sarcopenic elderly.

Currently, no cutoff values exist for these parameters in relation to OSO as a singular entity. The cutoff values for FFMI and SMMI, which represent whole-body muscle mass, would be useful for types of BIA without the ability to assess appendicular lean mass (appSMMI). Further, the cutoff value for BUA will be useful for the screening of bone density in populations that do not have built-in reference T-scores in some QUS devices. The advantage of the ROC curve was the ability to determine cutoff values based on obese people presenting both osteoporosis and sarcopenia at the same time. The curve was plotted against obese people without the combined disorder. This allowed us to see how much worse-off an individual would be without accounting for obesity as a factor (obesity is the common denominator). In the current study, it was revealed that obese people with osteosarcopenia were likely to have HGS equal to or less than 16.5 kg (Table 3), which was marginally lower than the cutoff proposed by AWGS for the Asian population (18.0 kg). Ilich et al. [9], studying 258 white postmenopausal women with age of 61.6 ± 7.4 years and ≥ 35% of body fat, found that obese women with OS had significantly lower HGS compared to obese women without OS. Reduced grip strength has been shown to lead to a greater propensity for fragility fractures and associated morbidity [24]. Therefore, women with OSO carry with it a higher risk for frailty and fracture risk. Further, the lower HGS strength found in the current study may indicate fat infiltration into both muscle and bone, impairing each tissue's physiology and functioning.

While it is recognized that whole-body MRI provides the most accurate measurement of skeletal muscle mass, its use is limited by inconvenience, affordability, and access. Even the gold-standard dual-energy X-ray absorptiometry (DXA) shares the same issues that limit its utility in field studies. Therefore, the BIA is a more convenient method to be used in field studies. In the current study, the whole-body skeletal muscle mass index (SMMI) of ≤ 8.2 kg/m^2^ was derived using the ROC curve (Figure 1). Interestingly, this value was greater than the cutoffs set by several Asian and Western populations: SMMI < 6.40 kg/m^2^ (China, [36]), SMMI < 6.20 kg/m^2^ (France, [37]), SMMI < 6.50 kg/m^2^ (Taiwan, [38]), SMMI < 6.68 kg/m^2^ (Spain, [39]), SMMI < 6.75 kg/m^2^ (USA, [40]), and SMMI < 5.22 kg/m^2^ (Mexico, [41]). It is important to note that these cutoff values were derived using two standard deviations (SD) below the mean value of young adults, rather than the ROC curve. Further, studies have shown that BMI and BFP are positively correlated with SMMI [42]. It is possible that the higher cutoff for SMMI derived from the ROC curve is due to the high BFP of the current sample group (BFP ≥ 32%).

It is also interesting to note that the cutoff value for BUA in the current study was similar to the cutoff proposed by Johansen, Evans, and Stone [18], 52.85 dB/MHz versus 54.0 dB/MHz. Johansen, Evans, and Stone [18] found that subjects with BUA below the 54 dB/MHz threshold value were shown to have low femoral neck BMD. The study, however, had used 2.5 SD below the mean of young adults as the method to derive the cutoff value. Young adults tend to have higher bone density compared to older adults, which likely raised the cutoff values. High bone density has been consistently found to be correlated with obesity. Therefore, it is possible that the similarity in cutoff values is due to the reference groups used in the studies (BMD young adults ∼ BMD obese population).

#### 8.4.2. Lowest Quintile (20th)

Separating data into quintiles is another method used to create cutoff points for a given population. A quintile is a statistical value of a data set that represents 20% of a given population. The first quintile represents the lowest fifth of data and the final quintile represents the final or last fifth of data. This is a good method to show the distribution of data. For example, in order to determine the distribution of wealth in society, a socioeconomic study conferred by the government may use this method to quantify the maximum amount of money a family could possess in order to belong to the lowest quintile of society. This maximum amount can then be used as a prerequisite for a family to receive a type of welfare or special government subsidy aimed to assist the people of less fortunate in the society. In the case of sarcopenia, researchers have used this method based on the same concept and had been widely accepted by several working groups for sarcopenia [11, 32, 43]. Using quintiles is a convenient way to represent data. However, it may not be the best way to categorize data when the exposure is not normally distributed. It is fair to note that, in the current study, SMMI and appSMMI were nonnormally distributed data (although FFMI, HGS, and BUA were all normally distributed). Another advantage of using this method is that data from a reference group is not required. Therefore, cutoff values can be determined for any desired parameters. A study by Jang et al. [31] found that the cutoff points using the lowest quintile of Korean rural older adults were < 5.2 kg/m^2^ for women in height-adjusted appSMMI, comparable to reports from other countries. A Taiwanese study using the same method found that the cutoff for decreased appSMMI was < 5.5 kg/m^2^ for women [44]. Another Korean study found that the cutoff values for appSMMI were < 5.4 kg/m^2^, HGS < 9.1 kg, and gait speed < 0.5 m/s using the same method [45]. In the current study, the cutoff for appSMMI derived from this method was < 5.4 kg/m^2^ (Table 3), comparable to the study in Taiwan and Korea. The cutoff for handgrip strength, however, was higher in the current study compared to the aforementioned Korean study (HGS < 16 kg (current study, Table 3) versus HGS < 9.1 kg [45]). The Korean study, which had compared their own cutoff values (derived using lowest quintile) with cutoff values recommended by the Foundation for the National Institutes of Health (FNIH) argued that using the lowest quintile method showed better predictive values in mortality than using the FNIH cutoffs. However, this argument is only based on one study.

#### 8.4.3. Two SD below the Mean Value of a Young Reference Group

Despite its limitations, the most used method to derive cutoff values is the 2 SD below the mean for a young reference population. Working group such as EWGSOP, in particular, recommends the use of this method in defining sarcopenia. This recommendation is based on the understanding that body composition may be affected by race and environmental factors such as diet and physical activity. Therefore, the reference population should derive from the same population of interest and represents people who are at peaked conditions, such as young adults (Table 2). In the current study, the cutoff values derived using this method were lower than those derived using previously mentioned methods (ROC curve and lowest quintile), including the standard cutoffs (Table 3). Considering the skeletal muscle mass, muscle strength, and bone density, cutoff points defined as 2SD lower than healthy young adults in this study were the following: SMMI < 6.7 kg/m^2^, appSMMI < 4.7 kg/m^2^, HGS < 11.4 kg, and BUA < 37.7 dB/MHz (Table 3). These cutoffs were also much lower than the cutoffs proposed by AWGS for the Asian population and other studies (Table 3). Height-adjusted appendicular skeletal muscle mass, in particular, was much lower than other Asian and Western studies which had used the same methods to derive the cutoff values, appSMMI ≤ 5.40 kg/m^2^ (AWGS) [11], appSMMI < 5.8 kg/m^2^ (Japan) [46], appSMMI < 5.07 kg/m^2^ (Korea) [47], appSMMI ≤ 5.50 kg/m^2^ (EWGSOP) [32], appSMMI ≤ 5.67 kg/m^2^ (IWGS) [43], and appSMMI ≤ 5.18 kg/m^2^ (Society of Sarcopenia, Cachexia and Wasting Disorders) [48]. However, three studies had produced similar findings: appSMMI < 4.72 kg/m^2^ (Mexico) [41], appSMMI < 4.82 kg/m^2^ (China) [36], and appSMMI < 4.4 kg/m^2^ (Korea) [49]. Despite its popularity, this method is not without limitations. Visser [50] argued that the current definition of sarcopenia refers to a state of deficiency in muscle mass and does not indicate muscle loss [50]. Various studies had shown a progressive loss of musculoskeletal health after the third decade of life and continue to lose 1-2% of muscle mass after the fifth decade and becoming more evidenced after the sixth [51]. Therefore, caution was advised when using young adults as a group of reference as they have not been exposed to the same factors that older people have experienced throughout their lives, in addition to the already natural progression of muscle loss due to aging. Perhaps, using healthy, community-dwelling elderly with high quality of life could reflect with greater precision the deficiency of muscle mass instead of the comparison with young population. Therefore, various studies on the causes of sarcopenia are taking into account factors beyond just the risk factors, such as physical inactivity, dietary intake, hormonal influence, and cytokine levels, recognizing the disorder as a geriatric syndrome [52].

### 8.5. Comparisons of Diagnostic Criteria and Corresponding Cutoff Values

This section compares the abovementioned statistical modeling methods and the corresponding cutoff values (Table 3) while highlighting the problems resulting from the lack of uniformity in diagnostic criteria. Our findings suggest that individuals with high adiposity may require alternative cut-points to define low bone mass and low muscle mass. Studies have found that the body has its own conserved regulatory mechanism or mechanostat that senses mechanical strain and adapts accordingly. The mechanostat functions as an adaptive mechanism to optimize bone mass and architecture based on prevailing mechanical strain. Changes in weight, due to altered mass, weightlessness (spaceflight), and hypergravity (modeled by centrifugation), induce an adaptive skeletal response [53]. Therefore, as an “adaptive” mechanism, a body with high adiposity will have a stronger core structure to support the extra weight (high bone density and muscle mass). Therefore, what is considered as “sufficient” for normal-weight individuals may not be so for people with high adiposity, hence why they may require alternative cut-points. Lenzi et al. [53] also raised the question of the accuracy of defining sarcopenia in obese individuals. The author states that, in obese individuals, the augmentation of muscle mass in parallel with body fat mass serves as a protective mechanism for obese individuals to sustain the increased fat mass. In other words, the increase of muscle mass in parallel with fat mass is a way for the body to protect itself by increasing the core structure to sustain the extra weight load. This is also described by Wolff's Law of bone formation and Davis' Law of soft tissue increment [54]. Wolff's law, developed by the German anatomist and surgeon Julius Wolff (1836–1902) in the nineteenth century, states that bone in a healthy person or animal will adapt to the loads under which it is placed [55]. Similarly, Davis's Law (developed by an American orthopedic surgeon named Henry Gasset Davis), which is a corollary to Wolff's Law, describes how soft tissue increases according to the manner in which they are mechanically stressed [56]. In the case of osseous tissue, this protective mechanism was theorized to occur up to a point (mechanostat limit) until there is an imbalance between muscle mass, excess body fat, and total body size, resulting in disproportionate ratios between muscle mass and excess fat mass, creating a situation where body weight exceeds that which the skeleton and muscle mass can support. Lenzi et al. [53] hypothesized that, despite the appearance of good muscle mass in obese people, it is likely not sufficient in proportion to the total body mass to prevent the onset of functional impairment and disability [53]. A study by Norshafarina et al. in 2013 [57], which categorized its study sample into the sarcopenic group and the nonsarcopenic group, found that overall skeletal muscle mass (SMMI) for the nonsarcopenic Malaysian women over the age of 60 was 8.24 ± 3.74 kg/m^2^ [56]. This value is similar to the current study's cutoff for OS (8.2 kg/m^2^, Table 3). This finding shows that the cutoff value of SMMI to define sarcopenia in obese individual is similar to the mean SMMI of women without sarcopenia, suggesting a discordance and a need for alternative cutoffs for the obese population. In the current study, the ROC curve method allowed us to derive cutoff values specifically for obese population by using the obese as the population of reference, instead of mixing people of normal weight into the reference group. This is why the ROC curve method was concluded to be the most appropriate method to use, out of the 3 statistical modeling methods tested in the current study. Out of the 3 sets of new criteria proposed in Table 5, Criteria 1 is recommended to be the new screening test criteria as it has the highest percentage of sensitivity to screen for OSO. Whenever there is a trade-off between sensitivity and specificity, sensitivity is prioritized over specificity to detect screening test criteria [16].

In the case of screening tools, often, they are chosen based on the means and objective of the researchers. For example, T-scores require time-consuming calculations, which may hinder their use. Therefore, in the case of QUS, a direct cutoff value for indicator such as the BUA may be preferred. In addition, other efficiency criteria are also to be taken into account such as the rapidity, simplicity, and easy administration of the screening tool use. This reflects the feasibility of these tools in clinical practice. In the current study, we proposed cutoff values suitable for obese population, using portable and cost-effective screening tools. However, it is important to note that these cutoffs are given as an indication, due to the study's limitations in its external validity. Although the BIA and QUS methods are reliable techniques for measuring body composition and bone mineral density, they are by no means proper diagnostic tools. The next step of the study will be to validate the cutoffs using dual-energy x-ray absorptiometry (DXA), and with a larger sample size. Nevertheless, the current study's comparison of various diagnostic and statistical modeling methods may bridge the gap in knowledge of osteosarcopenia and contribute towards a more evidence-based and less theoretical definition of the syndrome, not only in epidemiological research but also in health services.

## 9. Conclusion

Creating specific cutoff points for the high-risk group such as the obese population is important for accurate labeling and identification of low bone mass and low muscle mass, so that appropriate intervention can be instigated and reduce the risk of having an advanced musculoskeletal disorder in their later years. Uniformity in diagnostic criteria is also important to facilitate standardizations and comparisons of this disorder between countries. Lack of uniformity could adversely affect public health policies. For example, an underestimation or overestimation of the prevalence of a specific disorder could increase the risk of providing unnecessary treatment to a false-positive patient or depriving a false-negative patient of treatments. In the current study, we proposed cutoff values suitable for obese population, using portable and cost-effective screening tools. Osteosarcopenic obesity (OSO) is a prevalent musculoskeletal syndrome conferring an increased risk of falls, fractures, and hospitalizations. Findings from preliminary studies suggest that OSO could be a good target for translational research due to interconnected pathways illustrating a cross-talk among fat, bone, and muscle tissues. Currently, resistance exercise, high protein, vitamin D, calcium, and creatine intake are the only evidence‐based strategies to reduce the progression of osteoporosis and sarcopenia. More research in OSO is needed as the recognition of this syndrome is currently in its infancy. Increased awareness among geriatrics and gerontology healthcare professionals is important and must be included in the context of public health policies.

### 9.1. Limitations and Strengths

Due to the study's limitations in its external validity, it is important to note that these cutoffs are given as an indication. Although we used statistical inference techniques, biases may have been present, largely due to the participant selection process which was mainly composed of voluntary subjects. These subjects could be more health-conscious and be willing to undertake a 1-hour interview, including bone density, functional performance, and body composition assessments, compared to a random sample of the population. However, our findings may bridge the gap in knowledge of OSO and contribute to the effort in standardizing the diagnostic criteria for OSO and accurately determine the magnitude of the syndrome in the elderly. ROC curve method may be the best method to be used for deriving cutoff values for OS studies in overweight and/or obese populations.

## Figures and Tables

**Table 1:** Characteristic differences between OSO, OO, SO, OB, and NR participants (*n* = 141).

Variables	Osteosarcopenic obese (OSO, *n* = 8, 5.7%)	Osteopenic obese (OO, *n* = 6, 4.3%)	Sarcopenic obese (SO, *n* = 24, 17.0%)	Obese-only (OB, *n* = 82, 58.2%)	Normal (NR, *n* = 7, 5.0%)	*p* value
Age (years)	67.4 (8.4)	57.0 (4.1)	61.7 (9.5)	59.2 (6.7)^+^	55.0 (IQR 14.0)	**0.031** ^*∗*^
Years since menopause (years)	10.5 (6.6)	6.2 (3.8)	10.0 (IQR 18.0)	7.0 (IQR 9.0)	12.00 (8.8)	0.262 ^*∗∗*^
Height (cm)	145.3 (6.0)	154.9 (3.0)^+^	151.2 (5.2)	153.7 (5.9)^+^	156.5 (IQR 6.5)^*δ*^	**0.0001** ^*∗*^
Weight (kg)	50.1 (5.7)	70.9 (10.8)^+^	54.8 (6.4)^*β*^	69.5 (IQR 11.4)^*δ*^	53.5 (3.0)^*β*^	**0.0001** ^*∗*^
BMI (kg/m^2^)	23.8 (2.9)^ƚ^	29.6 (4.4)	24.0 (2.9)^ƚ^	29.1 (IQR 5.0)^*δ*^	21.1 (0.7)^*β*,ƒ^	**0.0001** ^*∗∗*^
WC (cm)	74.9 (5.8)	86.7 (6.8)	78.4 (8.3)^ƚ^	88.7 (IQR 13.0)^*δ*^	73.6 (3.9)^ƚ^	**0.0001** ^*∗*^
Body fat mass (kg)	19.6 (4.4)	32.0 (7.4)^*δ*^	22.6 (5.0)^*β*^	30.4 (IQR 9.5)^*δ*^	14.8 (2.6)^*β*^	**0.0001** ^*∗*^
BFP (%)	38.7 (4.9)	44.9 (4.7)	40.8 (4.7)	43.7 (5.8)	27.5 (4.2)^*β*, *δ*^	**0.0001** ^*∗*^
Trunk fat (%)	39.6 (5.2)	45.5 (3.4)	41.4 (4.7)	44.5 (IQR 7.3)	27.8 (4.4)^*β*, *δ*^	**0.0001** ^*∗*^
FFMI (kg/m^2^)	14.5 (0.8)^ƚ^	16.2 (1.9)	14.1 (0.9)^ƚ^	16.2 (IQR 1.7)^*δ*^	15.3 (1.0)	**0.0001** ^*∗∗*^
SMMI (kg/m^2^)	7.5 (0.5)^ƚ^	8.1 (IQR 2.4)^*δ*^	7.4 (0.5)^ƚ^	8.7 (IQR 1.1)^*δ*^	8.3 (0.6)^ƒ^	**0.0001** ^*∗∗*^
Appendicular SMMI (kg/m^2^)	5.1 (0.5)	6.1 (IQR 1.4)^*δ*^	5.3 (IQR 0.4)^ƚ^	6.5 (IQR 0.8)^*δ*^	6.5 (IQR 0.7)^*δ*^	**0.0001** ^*∗*^
HGS (kg)	17.3 (3.5)	19.2 (2.6)	18.2 (4.6)	20.3 (4.9)	26.4 (4.6)^*β*, *δ*^	**0.001** ^*∗*^
Sit-to-stand test in 30 sec (times)	11.0 (IQR 3.0)	12.7 (4.6)	11.7 (3.1)	11.8 (4.0)	12.4 (2.8)	0.974 ^*∗*^
Gait speed (m/s)	0.9 (0.3)	0.9 (0.2)	0.9 (0.3)	0.9 (IQR 0.4)	0.9 (0.2)	0.855 ^*∗*^
Balance (sec)	13.6 (12.5)	18.7 (8.3)	14.5 (IQR 21.0)	25.0 (IQR 17.0)	30.0 (IQR 7.0)	**0.046** ^*∗*^

All results are presented in mean (standard deviation) unless otherwise stated. Interquartile range (IQR) is presented with median. Fourteen participants (*n* = 14, 10%) are normal weight with either sarcopenic or osteopenic/osteoporotic. AppSMMI: appendicular skeletal muscle mass index; BMI: body mass index; BFP: body fat percent; FFMI: fat-free mass index; HGS: handgrip strength; SMMI: skeletal muscle mass index; WC: waist circumference; OSO: T-score ≤ −2.5, appendicular skeletal muscle mass index ≤ 5.7 kg/m^2^, and body fat percent ≥ 32%; OO: T-score ≤ −2.5, appendicular skeletal muscle mass index > 5.7 kg/m^2^, and body fat percent ≥ 32%; SO: T-score > −2.5, appendicular skeletal muscle mass index ≤ 5.7 kg/m^2^, and body fat percent ≥ 32%; normal: nonobese (percent body fat < 32%), nonsarcopenic (appSMMI > 5.7 kg/m^2^), and nonosteopenic/osteoporotic (T-score > −2.5).  ^*∗*^Analyzed using one-way ANOVA, with Tukey HSD post hoc test.  ^*∗∗*^Analyzed using one-way ANOVA (Welch test) with Games–Howell post hoc test due to the violation of the homogeneity of variances assumptions (Levene statistic *p* value < 0.05). ^+^Different from OSO. ^*δ*^Different from SO and OSO. ^*β*^Different from obese-only and OO. ^ƚ^Different from obese-only. ^ƒ^Different from SO. Normality tested using Shapiro–Wilks test: *p* value > 0.05 is normally distributed.

**Table 2:** Differences in characteristics: young versus postmenopausal women.

Variables	*N*	Young women Mean (SD)	*N* ^*ƚ*^	Postmenopausal women Mean (SD)	^*∗*^*p* value	Cohen's *d* (effect size)
Age (years)	118	22.1 (2.2)	118	60.0 (7.8)	**0.0001**	−6.574
Height (cm)	118	159.3 (5.5)	118	152.8 (6.2)	**0.0001**	1.104
Weight (kg)	118	56.9 (11.6)	118	63.9 (12.6)	**0.0001**	−0.586
BMI (kg/m^2^)	118	22.4 (4.5)	118	27.4 (5.3)	**0.0001**	−1.015
WC (cm)	106	71.9 (9.3)	115	84.8 (12.5)	**0.0001**	−1.171
BFP (%)	118	32.4 (7.7)	118	41.1 (7.6)	**0.0001**	−1.133
FFMI (kg/m^2^)	118	14.8 (1.5)	118	15.8 (1.7)	**0.0001**	−0.599
SMMI (kg/m^2^)	118	8.0 (0.9)	118	8.5 (1.1)	**0.0001**	−0.482
AppSMMI (kg/m^2^)	118	5.9 (0.7)	118	6.1 (0.9)	**0.05**	−0.248
HGS (kg)	117	24.7 (4.2)	112	19.8 (5.0)	**0.0001**	1.058
BUA (dB/MHz)	117	86.5 (16.3)	116	70.3 (17.2)	**0.0001**	0.970
SOS (m/s)	117	1570.9 (33.1)	116	1525.0 (31.9)	**0.0001**	1.415
Est. BMD (g/m^2^)	117	0.610 (0.122)	116	0.449 (0.124)	**0.0001**	1.310
QUI/Stiffness	117	108.1 (19.9)	116	82.3 (21.0)	**0.0001**	1.268
T-score (MY ref.)	117	−0.4 (1.1)	116	−1.3 (1.0)	**0.0001**	1.323
Z-score	117	−0.1 (1.0)	116	0.0 (1.0)	0.34	−0.131

^ƚ^Postmenopausal women who reported to have been diagnosed with musculoskeletal-related disorders were excluded from the analysis (i.e., osteoarthritis, rheumatoid arthritis, and osteoporosis). SD: standard deviation; CI: confidence interval; BMI: body mass index; WC: waist circumference; BFP: body fat percent; FFMI: fat-free mass index; SMMI: skeletal muscle mass index; appSMMI: appendicular skeletal muscle mass index; HGS: handgrip strength; BUA: broadband ultrasonic attenuation; Est. BMD: estimated bone mineral density; SOS: speed of sound; QUI: quantitative ultrasonic index; MY: Malaysia.  ^*∗*^Analyzed using independent *T*-test. Formula for Cohen's *d* = *t* √(*N*1+N2/N1*∗*N2), small = 0.2, medium = 0.5, and large = 0.8.

**Table 3:** Comparison of cutoff values according to different methods.

Variables	ROC curve (*N* = 90)	Lowest 20th percentile (*N* = 118)	2SD below young reference group (*N* = 118)	Standard cutoff
FFMI (kg/m^2^)	15.2	14.4	12.8	—
SMMI (kg/m^2^)	8.2	7.6	6.7	—
AppSMMI (kg/m^2^)	—	5.4	4.7	5.7^ƚ^
Handgrip strength (kg)	16.5	16.0	11.4	18.0^ƚ^
BUA (dB/MHz)	53.0	55.3	37.7	54.0^ƒ^
SOS (m/s)	1492.15	1501.4	1458.8	—
Est. BMD (g/cm^2^)	—	0.349	0.205	—
T-score (MY ref.)	—	−2.1	−3.5	−2.5^*δ*^

ROC: receiver operating characteristic; SD: standard deviation; FFMI: fat-free mass index; SMMI: skeletal muscle mass index; appSMMI: appendicular skeletal muscle mass index; BUA: broadband ultrasonic attenuation; SOS: speed of sound; Est. BMD: estimated bone mineral density; MY: Malaysia. ^ƒ^Johansen, Evans, and Stone, 1999. ^ƚ^Chen et al., 2014. ^*δ*^World Health Organization (WHO).

**Table 4:** Optimal cutoff, sensitivity, specificity, and area under the ROC curves in predicting osteosarcopenia in obese postmenopausal women.

	Cutoff	Sensitivity (%)	Specificity (%)	AUC	*p* value	95% CI
HGS	16.5	62.5	76.0	0.698	0.066	0.544 to 0.853
SMMI	8.2	87.5	90.2	0.966	0.0001	0.923 to 1.000
FMMI	15.2	75.0	91.5	0.946	0.0001	0.887 to 1.000
BUA	52.85	87.5	95.1	0.987	0.0001	0.966 to 1.000
SOS	1492.15	87.5	96.3	0.991	0.0001	0.975 to 1.000

HGS: handgrip strength; SMMI: skeletal muscle mass index; FFMI: fat-free mass index; BUA: broadband ultrasonic attenuation; SOS: speed of sound; AUC: area under curve; CI: confidence interval; ROC: receiver operating characteristic curve.

**Table 5:** Prevalence of OSO and its variations based on the new criteria and cutoff values (*N* = 141).

Criteria and cutoff values used to define OSO	OSO	OO	SO	OB	NR
	*N* (%)	*N* (%)	*N* (%)	*N* (%)	*N* (%)
*Old*					
*T*-score ≤ −2.5	8 (5.7)	6 (4.3)	24 (17.0)	82 (58.2)	7 (5.0)
AppSMMI ≤ 5.7 kg/m^2^					
BFP ≥ 32%					
*New (Criteria 1)*					
BUA ≤ 52.85 dB/MHz	12 (8.5)	5 (3.5)	39 (27.7)	64 (45.4)	5 (3.5)
SMMI ≤ 8.2 kg/m^2^					
BFP ≥ 32%					
Sensitivity (%)	100	83.33	100	78.0	71.4
Specificity (%)	97.0	100	87.0	100	100
*New (Criteria 2)*					
BUA ≤ 52.85 dB/MHz	7 (5.0)	10 (7.1)	29 (20.6)	74 (52.5)	5 (3.5)
FFMI ≤ 15.2 kg/m^2^					
BFP ≥ 32%					
Sensitivity (%)	87.5	100	100	90.2	71.4
Specificity (%)	100	97.0	95.7	100	100
*New (Criteria 3)*					
BUA ≤ 52.85 dB/MHz	5 (3.5)	3 (2.1)	15 (10.6)	46 (32.6)	4 (2.8)
SMMI ≤ 8.2 kg/m^2^					
HGS ≤ 16.5 kg					
BFP ≥ 32%					
Sensitivity (%)	62.5	50.0	62.5	56.1	57.1
Specificity (%)	100	100	100	100	100

The sensitivity (Sn) is defined as the probability of a positive test in a diseased person (Sn = true positive ÷ true positive/false negative). The specificity (Sp) is defined as the likelihood of a negative test in a person without the disease (Sp = true negative ÷ true negative/false positive). Fourteen participants (*n* = 14, 10%) are normal weight with either sarcopenic or osteopenic/osteoporotic. OSO: osteosarcopenic obesity; OO: osteopenic obesity; SO: sarcopenia obesity; OB: obese-only; NR: healthy, normal weight; appSMMI: appendicular skeletal muscle mass index; SMMI: skeletal muscle mass index; FFMI: fat-free muscle mass index; BUA: broadband ultrasonic attenuation; BFP: body fat percent; HGS: handgrip strength.

## Data Availability

The data presented in this study are openly available in FigShare at https://doi.org/10.6084/m9.figshare.13786684.v1.
